# Overexpression of DeltaFosB in nucleus accumbens mimics the protective addiction phenotype, but not the protective depression phenotype of environmental enrichment

**DOI:** 10.3389/fnbeh.2014.00297

**Published:** 2014-08-29

**Authors:** Yafang Zhang, Elizabeth J. Crofton, Dingge Li, Mary Kay Lobo, Xiuzhen Fan, Eric J. Nestler, Thomas A. Green

**Affiliations:** ^1^Center for Addiction Research, Department of Pharmacology and Toxicology, University of Texas Medical BranchGalveston, TX, USA; ^2^Anatomy and Neurobiology, University of Maryland School of MedicineBaltimore, MD, USA; ^3^Neuroscience, Mount Sinai School of MedicineNew York, NY, USA

**Keywords:** ∆FosB, environmental enrichment, depression, cocaine self administration, adeno-associated virus (AAV), overexpression

## Abstract

Environmental enrichment produces protective addiction and depression phenotypes in rats. ΔFosB is a transcription factor that regulates reward in the brain and is induced by psychological stress as well as drugs of abuse. However, the role played by ΔFosB in the protective phenotypes of environmental enrichment has not been well studied. Here, we demonstrate that ΔFosB is differentially regulated in rats reared in an isolated condition (IC) compared to those in an enriched condition (EC) in response to restraint stress or cocaine. Chronic stress or chronic cocaine treatment each elevates ΔFosB protein levels in the nucleus accumbens (NAc) of IC rats, but not of EC rats due to an already elevated basal accumulation of ΔFosB seen under EC conditions. Viral-mediated overexpression of ΔFosB in the NAc shell of pair-housed rats (i.e., independent of environmental enrichment/isolation) increases operant responding for sucrose when motivated by hunger, but decreases responding in satiated animals. Moreover, ΔFosB overexpression decreases cocaine self-administration, enhances extinction of cocaine seeking, and decreases cocaine-induced reinstatement of intravenous cocaine self-administration; all behavioral findings consistent with the enrichment phenotype. In contrast, however, ΔFosB overexpression did not alter responses of pair-housed rats in several tests of anxiety- and depression-related behavior. Thus, ΔFosB in the NAc the shell mimics the protective addiction phenotype, but not the protective depression phenotype of environmental enrichment.

## Introduction

Life experience, especially in the early stages of life, has a profound impact on the behavior of animals throughout life. Environment plays an essential role in the vulnerability and resistance to mental disorders in humans (Elisei et al., 2013; Akdeniz et al., 2014; Kato and Iwamoto, 2014; van Winkel et al., 2014). In rodent models, living in an enriched environment from weaning through young adulthood was reported to produce protective addiction and depression phenotypes (Green et al., 2002, 2003, 2010; Laviola et al., 2008; Solinas et al., 2008, 2009; El Rawas et al., 2009; Thiel et al., 2009, 2010). In this paradigm, animals are assigned to either an enriched condition (EC) in which animals are group-housed and have daily access to novel objects, or an isolated condition (IC) in which animals are single housed without novelty or social contact. Animals reared in the enriched condition, which includes social contact, exercise and novelty, exhibit less reinforcement and seeking of cocaine or amphetamine in the intravenous drug self-administration paradigm (Green et al., 2002, 2010). In addition to the addiction phenotype, such exposure to enrichment produces an antidepressant-like effect in animal models of depression (Green et al., 2010; Jha et al., 2011). Specifically, enriched animals exhibit decreased anhedonia-like behavior in the sucrose preference test, less social withdrawal in a social interaction test, and less immobility in the forced swim test (FST). Despite the anti-addiction and antidepressant-like effects of enrichment, the mechanisms underlying these protective phenotypes of environmental enrichment remain incompletely understood, although our prior research has implicated a role for decreased activity of the transcription factor, CREB, in the nucleus accumbens (NAc) in mediating some of the effects of environmental enrichment (Green et al., 2010; Larson et al., 2011). Thus, the goal of these differential rearing studies is to use a basic science approach to identify molecular mechanisms of resilience that can later be translated to the clinic. This approach is the environmental equivalent to well established genetic strategies such as selective breeding (McBride et al., 2014).

Here we focus on another transcription factor, ΔFosB, which is induced prominently in the NAc by certain forms of stress or by virtually all drugs of abuse, including cocaine, morphine, alcohol, nicotine, and amphetamine (Hope et al., 1992; Kelz and Nestler, 2000; Perrotti et al., 2004, 2008). As a transcription factor, ΔFosB dimerizes with Jun family proteins, preferentially JunD, to form an active AP-1 complex that binds to the AP-1 response element to enhance or repress the transcription of its target genes (Nestler, 2001), although new research suggests that ΔFosB can also act as a homodimer (Wang et al., 2012). The ΔFosB protein is a truncated splice variance of the *FosB* gene, which causes the ΔFosB protein to lack two C-terminal degron domains, preventing the ΔFosB protein from the rapid degradation seen with FosB and all other Fos-family proteins. Because ΔFosB is extraordinarily stable in the NAc, ΔFosB acts very differently in response to acute vs. chronic stimuli compared to other Fos proteins. With repeated exposure to drugs of abuse or stress ΔFosB protein gradually accumulates and persists for days to weeks, while FosB and other Fos proteins are induced for only a short time (hours) and develop an attenuated induction upon subsequent exposure (Nestler et al., 2001; Nestler, 2008). The importance of ΔFosB is not only that it is highly induced by drugs of abuse and stress, but that manipulation of ΔFosB in the brain has been shown to affect the behavior of animals. Selectively inducing ΔFosB in the dynorphin medium spiny neurons in adult mice increases locomotor sensitivity in response to acute and repeated cocaine, as well as the rewarding responses to cocaine in the conditioned place preference paradigm and reinforcement in the self-administration paradigm (Kelz et al., 1999; Kelz and Nestler, 2000; Colby et al., 2003). Although the protective addiction and depression phenotypes have been described in detail for environmentally enriched rats, a possible role for ΔFosB in mediating these protective phenotypes has not been fully evaluated. Previous studies of environmental enrichment showed that, compared to standard environment (SE), an enriched environment increases basal ΔFosB levels in both D1 and D2 medium spiny neurons of striatal regions in mice (Solinas et al., 2009; Lobo et al., 2013). In addition, enriched Wistar rats showed elevated ΔFosB positive cells in the NAc and prefrontal cortex compared to SE rats, suggesting a possible role of ΔFosB in the protective addiction phenotype to nicotine (Venebra-Muñoz et al., 2014). Furthermore, overexpressing ΔFosB throughout the striatum of mice increases daily wheel running, which may be analogous to the increased activity of rats in an enriched environment (Werme et al., 2002). In the current study, we hypothesized that: (1) environmental enrichment would increase accumulation of basal ΔFosB levels in the NAc; and (2) this accumulation of ΔFosB would contribute to the protective effects of environmental enrichment.

## Materials and methods

### Animals

For environmental enrichment, male Sprague-Dawley rats (Harlan, Houston, TX, USA) were randomly assigned to either EC or IC housing from postnatal day 21 to day 51. EC rats were group-housed (20 per cage) in a large metal cage (70 × 70 × 70 cm) with several hard plastic objects (children’s toys, plastic containers, PVC tubes, etc.). These objects were replaced with new objects and rearranged into a novel configuration daily. IC rats were singly housed in standard polycarbonate cages. Rats remained in these conditions throughout the experiments and all behavioral tests and biochemical tests began after 51 days of age (i.e., at least 30 days of enrichment/isolation). For the overexpression of ΔFosB, male Sprague-Dawley rats (Harlan, Houston, TX, USA) were obtained at size 225–250 g and pair-housed in standard polycarbonate cages before being stereotactically injected with an adeno-associated viral vector (AAV2) overexpressing ΔFosB with green fluorescent protein (GFP) or just GFP as a control (see below). Standard rat chow and water were freely available to all rats except during behavioral tests and food regulation. All rats were maintained in a controlled environment (temperature, 22°C; relative humidity, 50%; and 12 h light/dark cycle, lights on 600 h) in an Association for Assessment and Accreditation of Laboratory Animal Care (AAALAC) approved colony. All experiments conformed to the NIH Guide for the Care and Use of Laboratory Animals and the University of Texas Medical Branch Institutional Animal Care and Use Committee.

Environmental enrichment is a compound manipulation consisting of novelty, social contact and exercise. Pair housing provides social contact and thus represents an EC (see NIH Guide). Thus, the appropriate control group for a condition with novelty, social contact and exercise would be a group without novelty, social contact or exercise, the IC condition. IC rats show fewer signs of chronic stress than EC rats. Specifically, EC rats have enlarged adrenals (Mlynarik et al., 2004), blunted CORT responses (Stairs et al., 2011), attenuated immediate-early gene induction (Zhang et al., manuscript in preparation) and ΔFosB accumulation (Solinas et al., 2009; Lobo et al., 2013), all signs of chronic stress (Crofton et al., in review).

### Psychological stress

Enriched and isolated rats were placed into disposable soft plastic rodent restrainers (DecapiCone®, Braintree Scientific Inc., MA, USA) for 60 min for either 1 day (acute) or 9 days (repeated). For short-exposure mRNA tests, 30 rats (5 rats per group) were decapitated 30 min after the beginning of the last period of restraint stress, rat brains were extracted and the NAc was dissected for mRNA analysis. For immunohistochemistry, 12 rats were perfused with saline and 4% paraformaldehyde, brains extracted, post-fixed in 4% paraformaldehyde and stored in 20% glycerol in 1xPBS at 4°C. Rat brains were sliced at 40 μm with a freezing microtome. Brains were harvested 24 h after the final stress to allow the full-length FosB protein to degrade (Perrotti et al., 2008).

### Intravenous cocaine self-administration with environmental enrichment

#### Intravenous catheter implantation

Rats were anesthetized using ketamine (100 mg/kg IP) and xylazine (10 mg/kg IP) and a Silastic catheter was inserted and secured in the jugular vein, exiting the skin on the animal’s back. Each day the catheters were infused with 0.1 ml of a sterile saline solution containing heparin (30.0 U/ml), penicillin G potassium (250,000 U/ml) and streptokinase (8000 IU/ml) to prevent infection and maintain catheter patency throughout the duration of experiments.

#### Cocaine self-administration with environmental enrichment

Twenty enriched and 20 isolated rats were placed in operant chambers 30 × 24 × 21 cm (Med-Associates, St. Albans, VT) and allowed to press a lever for infusions of cocaine (0.5 mg/kg/infusion, NIDA drug supply, Research Triangle Institute, NC, USA) or saline under a fixed ratio 1 (FR1) schedule for 2 h per day for a total of 14 days. To maintain similar cocaine intake between the EC and IC groups there was a maximum of 30 infusions per session. Tissue processing capacity was limited to 30 samples, so the lowest-responding rats from each group were not processed, leaving Ns of 8 for cocaine and 7 for saline groups. Thus, there were no EC/IC differences in total cocaine intake or timecourse of infusions between EC and IC rats. Rat brains were extracted 3 h after the beginning of the last self-administration session and the NAc was dissected for mRNA and protein analysis. One side of the NAc was used for Western blot, the other side used for qPCR.

### Non-contingent cocaine administration with environmental enrichment

For direct comparison to previously published literature (Hope et al., 1994; Chen et al., 1995), EC (*N* = 12) and IC rats (*N* = 12) were injected with saline or 20 mg/kg cocaine intraperitoneally (IP) for 1 day (acute) or 9 days (repeated). One EC sample was lost during processing. The acute group received injections of saline for 8 days and one injection of cocaine on day 9 so that all rats received the same number of injections. Brains were extracted 30 min after the last injection and the NAc dissected for mRNA analysis.

### Quantification of mRNA using qPCR

The RNA was extracted by homogenizing in RNA STAT-60 (Teltest, Friendswood, TX), separating RNA from DNA and protein using chloroform, and precipitating the total RNA with isopropanol. Contaminating DNA was removed (TURBO DNA-Free, Life Technologies, CA, USA) and 5 ug of the purified RNA was reverse transcribed into cDNA (SuperScript III First Strand Synthesis: Invitrogen catalog # 18080051). ΔFosB mRNA was quantified using quantitative real-time PCR (SYBR Green: Applied Biosystems, Foster City, CA) on an Applied Biosystems 7500 fast thermocycler with primers designed to detect only ΔFosB (forward: AGGCAGAGCTGGAGTCGGAGAT; reverse: GCCGAGGACTTGAACTTCACTCG) and normalized to primers designed to detect rat GAPDH (forward: AACGACCCCTTCATTGAC; reverse: TCCACGACATACTCAGCAC). All primers were validated and analyzed for specificity and linearity prior to experiments (Alibhai et al., 2007).

### Western blot

The right side of the NAc from cocaine or saline self-administering EC and IC rats was homogenized in a buffer containing sucrose, Hepes buffer, sodium fluoride, 10% SDS, and protease and phosphatase inhibitors (Sigma-Aldrich: P-8340, P-2850, P-5726). Protein concentration was assessed using the Pierce BCA Protein Assay Kit (Thermo Scientific, IL, USA). Because protein extracted from one rat was not enough for analysis, 2 samples from the same group were pooled together, producing 4 samples for each group. Protein samples were denatured at 95° for 5 min and run on a 10–20% polyacrylamide gradient gel (Criterion TGX, Bio-Rad Laboratories, CA, USA) then transferred to a polyvinylidene fluoride (PVDF) membrane (Millipore, MA, USA). The membrane was blocked with blotting-grade blocker (nonfat dry milk), incubated with ΔFosB primary antibody (rabbit, 1:1000, #2251, Cell Signaling Technology, MA, USA) and β-actin primary antibody (mouse, 1:1000, Cell Signaling Technology, MA, USA), washed with TBST and then incubated with fluorescent secondary antibodies (donkey anti-rabbit (780 nm), donkey anti-mouse (680 nm), 1:15000, Li-Cor Biosciences, NE, USA). Western blots were then imaged (Odyssey, Li-Cor Biosciences, NE, USA) and protein levels quantified with the Odyssey software.

### Immunohistochemistry

**Figure 1:**
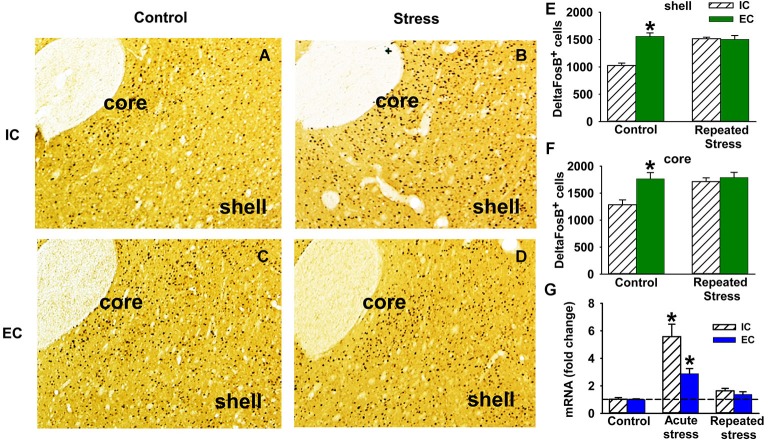
**Stress and ∆FosB in EC and IC rats. (A–D)** Representative immunohistochemistry DAB staining of ΔFosB in NAc shell and core of IC (**A** and **B**) and EC (**C** and **D**) rats with (**B** and **D**) and without (**A** and **C**) repeated stress (*N* = 3). **(E)** Quantification of the number of ΔFosB positive cells (±SEM) in NAc *shell* induced by repeated restraint stress in IC and EC rats. **(F)** Number of ΔFosB positive cells (±SEM) in NAc *core* induced by repeated restraint stress. **(G)** Fold change of ΔFosB ***mRNA*** (±SEM) induced by acute and repeated restraint stress in IC and EC rats (30 min; *N* = 5). Asterisks (*) denote significant difference from corresponding control.

For Figure 1 (*N* = 3), cells containing ΔFosB were visualized and counted through immunohistochemical labeling of ΔFosB in NAc slices stained with DAB (DAB peroxidase substrate kit, Vector Laboratories, CA, USA). The brains were extracted, post fixed, cryoprotected and sectioned into 40 μm slices containing the NAc on a sliding freezing microtome (Leica Biosystems, IL, USA). The slices remained floating and were rinsed with 1xPBS before endogenous peroxidases were quenched, prior to blocking with 3% normal goat serum (Jackson ImmunoResearch, PA, USA) with 0.3% triton and avidin D (Vector Laboratories, CA, USA). NAc slices were incubated with FosB primary antibody overnight (1:1000, Santa Cruz Biotechnology, Dallas, TX, USA) with 3% goat serum, 0.3% triton, 1xPBS, and biotin solution (Vector Laboratories, CA, USA). Although this antibody recognizes both FosB and ΔFosB, prior Western blot studies showed that at 24 h post stimulation, the vast majority of the immunohistochemical signal is composed of ΔFosB because FosB degrades well before 24 h (Perrotti et al., 2008). After washing, slices were incubated with a biotinylated goat anti-rabbit secondary antibody IgG (Vector Laboratories, CA, USA), goat serum, and 1xPBS. Then, slices were incubated with an avidin-biotin complex (ABC) peroxidase stain for 15 min (Thermo Scientific, IL, USA). Finally, slices were mounted, dehydrated using ethanol and CitriSolv (Fischer Scientific, MA, USA) and coverslipped with DPX (Fisher Scientific). For cell counting, sections were sampled from Bregma +1.80 to +1.44 from each animal. The total number of ΔFosB immunopositive cells was counted from four NAc sections from core and shell of each rat.

### Adeno-associated virus overexpressing ∆FosB

An AAV2-based vector that expresses ΔFosB and humanized renilla GFP (hrGFP; Winstanley et al., 2007, 2009a,b) or hrGFP control vector (*N* = 10 each) was injected bilaterally into the rat NAc. Because there are no IC humans, pair-housed rats were used instead of IC rats for this study to increase relevance to the scientific community by demonstrating the effects of ΔFosB *independent* of the EC/IC paradigm. An AAV expressing hrGFP but which does not overexpress ΔFosB was used as a control. The expression of ΔFosB *in vivo* was validated by immunofluorescence staining with FosB primary antibody (1:200, Rabbit, Cell Signaling Technology, MA, USA). AAV vectors were injected into the NAc shell bilaterally (1 μl/side over 10 min) using coordinates (*AP* = 1.7, *L* = 2.0, *D* = −6.5). The behavioral tests started 3 weeks after the stereotaxic surgery. Accurate placement was determined immunohistochemically after the conclusion of behavioral testing.

### Sucrose neophobia

ΔFosB overexpressing rats (*N* = 10) and control rats (*N* = 8) were handled for 1 week prior to the start of behavior tests. To test for anxiety-like behavior, rats were evaluated for neophobia to a novel taste (sucrose). Rats were separated into individual cages and water was removed at 1600 h. Standard rat water bottles were filled with a 1% w/v sucrose solution in the rats’ normal “tap” water and weighed before being placed on each cage at 1800 h. After 30 min the bottles were removed and re-weighed, and the difference of the weight of sucrose bottles before and after the test was calculated. Then, sucrose was replaced on the cages for an additional 2 days to let the rats familiarize to the flavor of sucrose prior to the sucrose preference test.

### Elevated plus maze

Another test of anxiety-like behavior, the elevated plus maze (EPM), was tested 2 days after sucrose neophobia. The EPM measures vector-modified exploratory behavior in a novel and anxiety-producing environment (Green et al., 2008). Two closed arms and two open arms (Med Associates Inc., VT, USA) measuring 12 × 50 cm were 75 cm above the floor and had photobeams at the entrance of each arm. Time spent on the open arms was monitored for 5 min by photobeam breaks using Med-PC software.

### Cold stress-induced defecation

On the day after EPM, a third anxiety test was used: defecation in response to a mildly stressful environment (cold). Polycarbonate mouse cages (33 × 17 × 13 cm) were pre-chilled on ice for 10 min. The rats were placed in the cages on ice for 30 min and the number of fecal boli was recorded every 5 min.

### Social contact

On the following day, depression-like behavior was measured using a social interaction test. Rats were separated for the 24 h prior to testing. On test day the rats were placed in a novel environment (plastic container, 45 × 40 × 45 cm) with their cage mate and behavior was video recorded for 30 min. The amount of time the pair of rats spent grooming each other was measured by an investigator blind to the rats’ condition.

### Sucrose preference

After social contact, the sucrose preference test was used as a model of anhedonia. Pair-housed rats were separated at 1600 h with food but not allowed access to water for 2 h. At 1800 h two pre-weighed water bottles were placed on each cage, one containing water, the other a 1% sucrose solution in water. The water bottles were placed in the normal position while the sucrose was placed approximately 10 cm away. The bottles were removed and re-weighed after 15 min.

### Locomotor activity

Three days after sucrose preference, locomotor activity was assessed under normal light conditions by placing the rats in clear Plexiglas chambers (40 × 40 × 40 cm) with a thin layer of bedding, surrounded by two 4 × 4 photobeam matrices, one 4 cm above the ground and one 16 cm above the ground to record horizontal ambulation and vertical (rearing) activity. Photobeam breaks were monitored for 2 h by a modified open field activity system (San Diego Instruments, CA, USA).

### Forced swim test

The last spontaneous behavioral test was the FST, a model sensitive to antidepressants. Rats were placed into a Plexiglas cylinder filled with approximately 14 L of room temperature (24 ± 0.5°) water for 15 min on Session 1, and 5 min on Session 2 the following day. The rats were dried and placed back into their home cages. Swimming activity was video recorded and the latency to the first period of immobility (1 s) and total time immobile were determined for Session 2 by an investigator blinded to the conditions.

### Sucrose operant responding

Control AAV rats and ΔFosB-overexpressing rats were regulated to 85% of free-feeding weight over 7 days. All rats were trained to bar press for sucrose pellets (Bio-Serv, NJ, USA) on a FR1 schedule of reinforcement for 15 min sessions on 5 consecutive days. Rats were then given free access to food for 3 days and again allowed to bar-press for sucrose pellets on an FR1 schedule for 15 min, this time at 100% free-feed weight.

### Cocaine self-administration

#### Acquisition

One week after catheter surgery (as described above), all rats (7 control rats and 10 ΔFosB overexpressing rats, one control rat was lost from catheter surgery) were placed in operant chambers 30 × 24 × 21 cm (Med-Associates, St. Albans, VT) and allowed to self-administer 0.2 mg/kg/infusion unit dose of cocaine for 2 h per session for 4 days; then 0.5 mg/kg/infusion for 3 days on an FR1 schedule. Each infusion was delivered intravenously in a volume of 0.01 ml over 5.8 s. The infusion was signaled by illumination of two cue lights for 20 s, which signaled a timeout period during which no further infusions could be attained.

#### Extinction

Because chronic cocaine exposure would presumably induce accumulation of ΔFosB in control rats, which would cause the rats in both vector conditions to have high levels of ΔFosB in the brain, the rats were confined to their home cages for 4 days without self-administration to allow ΔFosB protein levels to decrease in control-vector rats. After 4 days abstinence, rats were placed in the operant chamber and allowed to self-administer saline instead of cocaine under an FR1 schedule for 1 h sessions for 3 consecutive days.

#### Fixed ratio dose response

Each rat (control and ΔFosB overexpressing) was allowed to self-administer 0.00325, 0.0075, 0.015, 0.03, 0.06, 0.125, 0.25, 0.5 mg/kg/infusion cocaine in ascending order on an FR1 schedule each day for 5 consecutive days. Rats self-administered each dose of cocaine for 30 min.

#### Cocaine-induced reinstatement

Rats underwent a within-session reinstatement procedure. Rats received 0.5 mg/kg/ infusion on an FR1 schedule for 60 min followed by 3 h of extinction (with contingent cocaine cues). Next they received an IP injection (Green et al., 2010) of cocaine of one of five doses (0, 2.5, 5, 10, or 20 mg/kg) in a random order for each rat across the 5 sessions of reinstatement. The last 3 h phase of the session was reinstatement responding, again with cocaine cues but still without cocaine infusions. After each cocaine-induced reinstatement session, the rats received 2 intervening days of high dose (0.5 mg/kg/infusion) cocaine on an FR1 schedule for 2 h to maintain high rates of responding across sessions. During the cocaine self-administration process, the catheters of some rats gradually lost patency; hence, the data of 6 control rats and 7 ΔFosB-overexpressing rats were used in this analysis.

### Statistical analysis

Two-way analyses of variance (ANOVAs) and two-way repeated-measures ANOVAs were done to compare the four treatment groups and planned comparisons were used to compare differences among conditions. Significance between only two conditions was analyzed using a Student’s *t*-test. All *t*-test data passed the Shapiro-Wilk test of normality. All data are expressed as mean ± SEM. Statistical significance was set at *p* < 0.05. All of the enriched rats for a single experiment were housed in one cage but treated as separate subjects, providing implications regarding the issue of potential pseudoreplication.

## Results

### EC rats display higher basal levels of ∆FosB in NAc than IC rats

**Figure 2:**
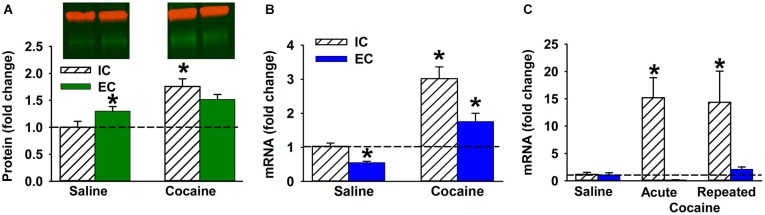
**Cocaine and ∆FosB in EC and IC rats. (A–B)** Mean ΔFosB protein **(A)** and mRNA **(B)** level (±SEM) in NAc after 14 days of saline or cocaine self-administration in IC and EC rats (*N* = 7–8). Red bands in Panel a denote β-actin used for normalization; green denotes ΔFosB protein. **(C)** Fold change of ΔFosB ***mRNA*** (±SEM) induced by acute or repeated IP cocaine injection in IC and EC rats (30 min; *N* = 4). Asterisks (*) denote significant difference from corresponding control.

Compared to IC rats, EC rats have a significantly higher number of ΔFosB positive cells in both NAc core (*t*_(4)_ = −3.31, *p* < 0.05) and shell (*t*_(4)_ = −6.84, *p* < 0.05) (Figures 1A,C,E,F), suggesting that the basal tone of ΔFosB is higher in EC rats compared to IC rats. In addition, Western blot results showed a strong trend for EC saline rats having higher basal level of ΔFosB protein in the NAc compared to IC saline rats (*t*_(6)_ = −2.03, *p* = 0.089; Figure 2A) using a two-tailed test. However, given the increased expression in Figures 1A–F and the increases seen in other papers (Solinas et al., 2009), we are confident in this effect. The Western blot findings also verify that virtually all of the FosB-like immunoreactivity detected by immunohistochemistry was ΔFosB and not FosB, which was not detectable at 24 h.

### ∆FosB is differentially induced in EC and IC rats by stress

There was a significant main effect of repeated restraint stress in both shell (*F*_(1, 8)_ = 16.6, *P* < 0.005) and core (*F*_(1, 8)_ = 7.9, *P* < 0.05) of the NAc and a main effect of environmental enrichment in shell (*F*_(1, 8)_ = 22.3, *P* < 0.005; Figures 1A–F). More importantly, the interaction between stress and environmental enrichment was also significant in both shell (*F*_(1, 8)_ = 25.6, *P* < 0.01) and core (*F*_(1, 8)_ = 6.7, *P* < 0.05). The interaction was such that, after repeated restraint stress, the number of ΔFosB positive cells significantly increased in IC rats, while this number did not change in EC rats after repeated stress.

To further investigate how ΔFosB is dynamically regulated by acute vs. repeated stress and to allow comparison with prior research (Alibhai et al., 2007), induction of ΔFosB ***mRNA*** was studied with acute and repeated restraint stress (Figure 1G). There was a significant main effect of stress (*F*_(2, 24)_ = 31.9, *P* < 0.001) and environmental enrichment (*F*_(1, 24)_ = 5.1, *P* < 0.05). In the IC rats, ΔFosB mRNA was strongly induced after acute restraint stress. However, with repeated stress, the induction of ΔFosB mRNA was significantly attenuated compared to the acute induction. There was also a significant interaction (*F*_(2, 24)_ = 4.6, *P* < 0.05), demonstrating that the acute induction of ΔFosB mRNA was less in EC rats compared to IC rats. Thus, EC rats have higher basal levels of ΔFosB *protein* in the NAc, but less ΔFosB *mRNA* induction in response to an acute stressor.

### ∆FosB is differentially induced by cocaine in NAc of EC and IC rats

To determine whether EC and IC rats respond differently to cocaine, we studied the regulation of ΔFosB protein and mRNA in rat NAc after cocaine self-administration (Figures 2A,B respectively). A Western blot revealed a significant main effect of cocaine (*F*_(1, 12)_ = 24.9, *P* < 0.001) and a significant interaction (*F*_(1,12)_ = 5.5, *P* < 0.05). The interaction was such that ΔFosB increased more in IC rats than EC rats (Figure 2A). In fact, after cocaine self-administration ΔFosB protein levels were significantly elevated *only* in IC rats. Regarding mRNA levels, qPCR results also revealed a significant main effect of cocaine (*F*_(1, 26)_ = 47.1, *P* < 0.001) and main effect of environmental enrichment (*F*_(1, 26)_ = 13.8, *P* < 0.005). Although overall levels were lower in EC rats, both groups increased ΔFosB mRNA (Figure 2B).

Although the protein data supported the original hypothesis, it was hypothesized from Figure 1G that EC rats would show less *mRNA* induction than isolated rats in the above cocaine experiment, which did not happen, likely because Figure 1G utilized a 30 min timepoint and the cocaine experiment utilized a 3 h timepoint. To further interrogate the mRNA hypothesis, a 30 min time point was used to explore both acute and repeated cocaine treatment as a better comparison to Figure 1G. Because acute cocaine self-administration is problematic by nature (i.e., acquisition learning), EC and IC rats were given acute or 9 days of repeated non-contingent cocaine IP injections (20 mg/kg). As hypothesized, there was a significant main effect of environmental enrichment (*F*_(1, 17)_ = 14.3, *P* < 0.005), but the cocaine treatment main effect (*F*_(2, 17)_ = 3.4, *P* = 0.057) and the interaction (*F*_(2, 17)_ = 3.4, *P* = 0.055) only showed strong trends with a two-tailed test. However, given that we had directional hypotheses from Figure 1G, we are extremely comfortable in our opinion that EC rats display less induction than IC rats (Figure 2C).

### Overexpression of ∆FosB in NAc shell mimics the protective enrichment-induced addiction phenotype

**Figure 3:**
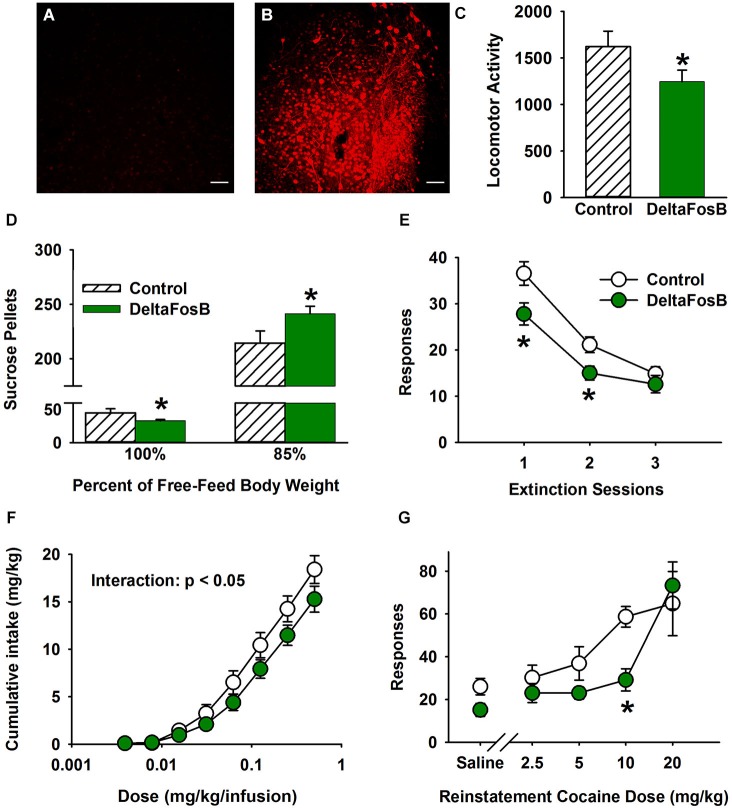
**Overexpression of ∆FosB in NAc shell mimics the protective addiction phenotype of environmental enrichment. (A–B)** Representative immunohistochemistry of ΔFosB for hrGFP control **(A)** and ΔFosB-overexpressing **(B)** AAV vectors. The bar represents 50 μm **(C)** Effect of overexpression of ΔFosB on locomotor activity. **(D)** Effect of overexpression of ΔFosB on sucrose pellet operant responding under hunger-motivated (85% free feed body weight) and non-hunger motivated conditions (100% free feed weight). **(E)** Effect of overexpression of ΔFosB on extinction of cocaine self-administration. **(F)** Effects of overexpression of ΔFosB on cumulative cocaine self-administration: FR dose response function. **(G)** Overexpression of ΔFosB significantly decreased the reinstatement response to cocaine injections. *N* = 6–10 for all behavior experiments. Asterisks (*) denote differential responding from control vector group.

To investigate the effect of ΔFosB on rat behavior independent of environmental enrichment/isolation (i.e., to make these results more relevant to non-EC/IC studies), adeno-associated virus (AAV) was used to overexpress ΔFosB bilaterally in the NAc in non-enriched pair-housed rats. According to our previous studies, the NAc shell is most sensitive to control depression-related and drug taking/seeking behavior so AAV vectors were injected in the NAc shell in this study (Green et al., 2006, 2008, 2010). Figures 3A,B show the representative immunohistofluorescence of ΔFosB with the control vector (panel A; i.e., endogenous ΔFosB expression) and the ΔFosB-overexpressing vector (panel B) in the NAc shell.

Having validated the titer, *in vivo* expression and general placement of the viral vector, we first studied the effect of overexpression of ΔFosB in anxiety models. Overexpression of ΔFosB in the NAc shell was not sufficient to reproduce the anxiogenic effect of environmental enrichment in the sucrose neophobia and cold-stress induced defecation paradigms (data not shown). In addition, there was no effect on the EPM (data not shown). Because environmental enrichment produces an antidepressant-like effect in rats, we next conducted depression-related tests on ΔFosB-overexpressing rats. Similar to anxiety models, results showed that overexpressing ΔFosB in NAc shell was not sufficient to decrease depression-like behavior in the sucrose preference test, the social interaction test, or the FST (data not shown).

In the environmental enrichment paradigm, EC rats exhibit lower basal locomotor activity than IC rats (Bowling et al., 1993; Bowling and Bardo, 1994; Smith et al., 1997; Green et al., 2003, 2010). To investigate the effect of overexpressing ΔFosB in the NAc shell, spontaneous locomotor activity was tested for 120 min. Using a two-tailed test the results revealed that overexpressing ΔFosB in the NAc shell produced a strong trend for decreased basal locomotor activity in rats (Figure 3C; *t*_(16)_ = 1.84, *p* = 0.084). Despite not being quite statistically significant with a two-tailed test, these data are still intriguing given that they conform to our explicit directional hypothesis based on Green et al. (2010), which is consistent with the effect of environmental enrichment.

In contrast to depression and anxiety models, overexpression of ΔFosB in NAc shell was able to produce an EC-like phenotype in multiple addiction/reinforcement paradigms. In the sucrose pellet operant self-administration test, there was a significant interaction between ΔFosB overexpression and hunger motivation of rats (*F*_(1, 16)_ = 7.4, *P* < 0.01). Rats overexpressing ΔFosB in the NAc shell took significantly *more* sucrose pellets under hunger-motivated conditions (i.e., at 85% free feed bodyweight), but fewer pellets under the low motivated condition (i.e., 100% free feed weight; Figure 3D), which mimics the EC phenotype perfectly (Green et al., 2010).

In the environmental enrichment paradigm, EC rats displayed reduced cocaine-seeking behavior in extinction and cocaine-induced reinstatement (Green et al., 2010). Thus, cocaine-taking and seeking behavior was measured in ΔFosB-expressing rats using the intravenous cocaine self-administration paradigm. As a model of craving, the cocaine extinction paradigm revealed that ΔFosB overexpression in the NAc shell decreased drug-seeking behavior (*F*_(1, 15)_ = 6.7, *P* < 0.05; Figure 3E). There was also a significant main effect of session (*F*_(2, 30)_ = 74.0, *P* < 0.001). For maintenance responding under an FR1 schedule, there was a significant main effect of dose (*F*_(7, 105)_ = 222.6, *P* < 0.001) and a significant interaction (*F*_(7, 105)_ = 2.3, *P* < 0.05) in cumulative cocaine intake. The nature of the interaction was such that differences were apparent only at higher doses of cocaine (Figure 3F). Finally, in cocaine induced reinstatement there was a significant main effect of dose (*F*_(4, 44)_ = 15.5, *P* < 0.001) and a trend for a main effect of ΔFosB overexpression using a two-tailed test (*F*_(1, 11)_ = 4.1, *P* = 0.067). However, given the directional hypothesis from Green et al. (2010) and the statistically significant and consistent results in Figures 3D,E,F, it is likely that ΔFosB decreases reinstatement (Figure 3G). Responding at the 10 mg/kg dose was significantly lower for ΔFosB-expressing rats. The results as a whole indicate that overexpressing ΔFosB in rat NAc shell decreases cocaine taking and seeking behavior, which is consistent with the behavioral effects of environmental enrichment.

## Discussion

The vulnerability of individuals to addiction and depression is heavily affected by environmental factors. Environmental enrichment is a paradigm that manipulates the living environment of animals, producing protective effects against many psychiatric conditions. ΔFosB plays a key role in regulating reward function in multiple brain regions, including the NAc and dorsal striatum (Koob et al., 1998; Wise, 1998; Wallace et al., 2008; Grueter et al., 2013; Pitchers et al., 2013). In this project, we studied the dynamic regulation of ΔFosB by restraint stress and cocaine in enriched and isolated rats. The major findings of this project are: (1) EC rats have elevated ΔFosB levels in the NAc at baseline compared to IC rats; (2) only IC rats accumulate additional ΔFosB protein with repeated stress; (3) EC rats display attenuated induction of ΔFosB mRNA subsequent to stress or cocaine; and (4) overexpressing ΔFosB in the NAc of pair-housed rats mimics the protective addiction phenotype, but not the protective depression phenotype.

One might expect from the published literature, which shows that transgenic ΔFosB overexpressing mice show increased sensitivity to cocaine reward and self-administration at low drug doses (Kelz et al., 1999; Colby et al., 2003; Vialou et al., 2010; Robison et al., 2013), that the ΔFosB-overexpressing rats in the current experiment would display increased propensity for cocaine self-administration and seeking. In the current experiments, however, overexpressing ΔFosB in the NAc shell decreased cocaine intake and cocaine seeking during extinction and reinstatement, indicating reduced motivation for cocaine. The discrepancy could be due to the fact that the transgenic mice expressed ΔFosB throughout the entire striatum, but only in dynorphin+ cells (Colby et al., 2003). In the current experiment ΔFosB was overexpressed through an AAV vector which infects dynorphin+ and enkephalin+ neurons. Second, the current study focused on the NAc shell rather than the whole striatal region.

In addition to the addiction phenotype, environmental enrichment produces antidepressant and anxiogenic-like profiles in rats (Green et al., 2010; Vialou et al., 2010). In the current study, overexpression of ΔFosB in the NAc failed to produce effects in any of the three depression or three anxiety tests. Although there are many possible factors that may contribute to ΔFosB mimicking the enrichment addiction but not the depression phenotype, it is possible that the NAc shell is more dominant for addiction-related behavior whereas depression-related behavior may be mediated more robustly by other regions. The present findings are at odds with studies in *mice* where ΔFosB overexpression in the NAc (where one cannot reliably distinguish shell vs. core) produced robust-antidepressant-like effects in several behavioral assays (Vialou et al., 2010). One possible reason is that it may be easier to see the effect of ΔFosB on severe stress behavioral models like social defeat stress. The current overexpression study investigated depression-like behavior in the absence of a severe stressor.

Consistently throughout this study, high basal levels of ΔFosB (e.g., from enrichment, repeated stress or cocaine) correlated with weaker subsequent induction of ΔFosB. This may represent a ceiling effect, with no further induction possible on top of elevated basal levels of the protein. It is also possible that accumulated levels of ΔFosB might feed back to inhibit further induction of ΔFosB mRNA after stress or cocaine as a negative feedback loop. For example, EC rats had high levels of ΔFosB and showed an attenuated induction of ΔFosB after stress or cocaine. This underscores the negative correlation between ΔFosB protein levels and its mRNA induction. The negative feedback of accumulated ΔFosB also accounts for the attenuated induction of ΔFosB with repeated stress in IC rats.

To be clear, we do not make any claims that the environmental enrichment paradigm has direct translational relevance, as there are very few children raised in true deprivation (it should be noted that socio-economic deprivation does not equate to environmental deprivation). The utility of this paradigm is that it is a non-drug, non-surgical, non-genetic manipulation that produces protective behavioral phenotypes for addiction and depression that can be exploited in a laboratory-controlled environment as a basic science tool for identifying molecular mechanisms underlying resilience to psychiatric conditions. Prior research has described the behavioral phenotypes in detail (Bowling et al., 1993; Bowling and Bardo, 1994; Bardo et al., 1995; Green et al., 2002, 2003; El Rawas et al., 2009) and more recent studies (Solinas et al., 2009; Green et al., 2010; Lobo et al., 2013), along with the current study, are providing clues as to the transcriptional mechanisms underlying these behavioral phenotypes. The downstream transcriptional target genes/proteins producing the protective phenotypes are currently being investigated (Fan et al., 2013a,b; Lichti et al., 2014).

Our conceptualization of environmental enrichment is that enrichment is a continuum with isolation at the low end and full enrichment at the high end. “Full” enrichment in this case is defined as an environment where the subjects are exposed to novelty, non-threatening social contact with conspecifics, and are allowed space and objects for exercise. These three factors all represent the compound condition of “enrichment” because they are each rewarding and each release dopamine in the NAc, and as such, activate a common neurobiological circuitry (Louilot et al., 1986; Calcagnetti and Schechter, 1992; Crowder and Hutto, 1992; Rebec et al., 1997; Bevins et al., 2002). In this conceptualization, isolation is considered the control group because it represents the absence of the manipulation (i.e., enrichment; Crofton et al., in review). However, other conceptualizations are possible. In one alternate conceptualization, the continuum is the same, but the isolation group is the experimental group and the enriched group is the control. In this model, depriving the subjects of normal enrichment *is* the actual manipulation. In this case, instead of saying that enrichment is protective, one would say that isolation confers susceptibility. Still a third conceptualization posits that there is no continuum and that enrichment and isolation are two fundamentally different manipulations. In this view, enrichment and isolation should be separated and both compared to a pair-housed control. Lack of a universal consensus as to the nature of enrichment represents a limitation of the paradigm, yet provides direction for future studies. Regardless, the results of these experiments stand firm regardless of the subsequent interpretation.

Environment and life experiences have a strong influence on the development and expression of many psychiatric conditions. Understanding the mechanism of the protective addiction and depression phenotypes of environmental enrichment addresses a fundamental question in mental disorder research—namely the environmental contribution to susceptibility or resilience to psychiatric conditions. This study underscores the significance of ΔFosB in regulating addiction-related behaviors. In future studies, the action of ΔFosB and its activating and inhibitory effects on specific target genes needs to be further explored within the environmental enrichment model.

## Funding and disclosure

Yafang Zhang, none; Elizabeth J. Crofton, none; Dingge Li, none; Mary Kay Lobo, none; Xiuzhen Fan, none; Eric J. Nestler, *R37DA007359*; Thomas A. Green, *DA029091*.

## Conflict of interest statement

The authors declare that the research was conducted in the absence of any commercial or financial relationships that could be construed as a potential conflict of interest.
